# Myelinoclastic diffuse sclerosis (Schilder’s disease) is immunologically distinct from multiple sclerosis: results from retrospective analysis of 92 lumbar punctures

**DOI:** 10.1186/s12974-019-1425-4

**Published:** 2019-02-28

**Authors:** S. Jarius, J. Haas, F. Paul, B. Wildemann

**Affiliations:** 1 Molecular Neuroimmunology Group, Department of Neurology, University of Heidelberg, Heidelberg, Germany; 2 NeuroCure Clinical Research Center, Charité – Universitätsmedizin Berlin, Berlin, Germany; 3 Department of Neurology, Charité – Universitätsmedizin Berlin, Berlin, Germany; 4 Clinical and Experimental Multiple Sclerosis Research Center, Department of Neurology, Charité – Universitätsmedizin Berlin, Berlin, Germany; 5 Experimental and Clinical Research Center, Max Delbrueck Center for Molecular Medicine, Charité Universitätsmedizin Berlin, Berlin, Germany; 6 Otto Meyerhof Center, Im Neuenheimer Feld 350, 69120 Heidelberg, Germany

**Keywords:** Schilder’s disease, Myelinoclastic diffuse sclerosis, Encephalitis periaxialis Schilder, Multiple sclerosis, Tumefactive, Demyelination, Central nervous system, Neuromyelitis optica, Baló’s concentric sclerosis

## Abstract

**Background:**

Myelinoclastic diffuse sclerosis (MDS; also termed Schilder’s disease) is a rare inflammatory demyelinating disorder of the central nervous system characterised by demyelination of vast areas of the white matter. It is unclear whether MDS is a variant of multiple sclerosis (MS) or a disease entity in its own right.

**Objective:**

To compare the cerebrospinal fluid (CSF) features of MDS with those of MS.

**Methods:**

Retrospective analysis of the CSF profile of all patients with MDS reported in the medical literature between 1960 and 2018.

**Results:**

The most striking finding was a substantial lack of oligoclonal bands (OCBs) in MDS, which were absent in at least 77% (30/39) of all lumbar punctures (LP) in the total cohort and in 86% in the subgroup of patients with normal very long-chain fatty acid serum ratios (VLCFA). Almost all cases published in the past 15 years were negative for OCBs. These findings are in contrast to MS, in which OCBs are present in up to 98% of cases (*p* < 0.00001 when compared with reference works in MS; both in adult and in pediatric patients). CSF pleocytosis was absent in at least 79% (46/58) of all LP (*p* < 0.0001 vs. MS) and in 92% (24/26) of LPs in the VLCFA-tested subgroup. CSF total protein levels were elevated in 56% of all LPs (*p* < 0.0001 vs. MS) and in 63% of LPs in the VLCFA-tested subgroup and were often higher than in typical MS (> 100 mg/dL in 13/22; up to 220 mg/dL). EBV serum antibodies, which are present in virtually all patients with MS, and the so-called MRZ (measles/rubella/zoster) reaction, a highly specific marker of MS, were absent in all of the few patients tested. In addition, we discuss further differences between MS and MDS, taking into account also Schilder’s original comprehensive case description from 1912.

**Conclusion:**

In the majority of patients diagnosed with MDS, CSF features differ significantly from those typically found in MS and are more similar to those previously reported in patients with myelin oligodendrocyte glycoprotein-immunoglobulin G (IgG)-positive encephalomyelitis, aquaporin-4-IgG-positive neuromyelitis optica spectrum disorders or Baló’s concentric sclerosis. Our data suggest that MDS and MS are immunopathologically distinct entities in the majority of cases.

## Background

Myelinoclastic diffuse sclerosis (MDS; also termed Schilder’s disease or encephalitis periaxialis diffusa) was first described in 1912 by the Austrian psychiatrist Paul Ferdinand Schilder (1886–1940) [1]. MDS is characterized by one or two extensive, often bilateral and roughly symmetrical demyelinating white matter lesions in the centrum semiovale. However, its exact nosological relationship to multiple sclerosis (MS) has remained elusive.

In a number of recent studies, we have demonstrated substantial differences in cerebrospinal fluid (CSF) features between patients with MS and patients with other autoimmune disorders of the central nervous system (CNS). In particular, we have found significantly lower frequencies of CSF-restricted oligoclonal bands (OCBs) and other markers of intrathecal immunoglobulin G (IgG) synthesis in patients with aquaporin-4 (AQP4)-IgG-positive neuromyelitis optica (NMO) spectrum disorders (NMOSD), myelin oligodendrocyte glycoprotein antibody-associated encephalomyelitis (MOG-EM), acute demyelinating encephalomyelitis (ADEM) and paraneoplastic neurological disorders (PND) than in MS, as well as significant differences in intrathecal IgG composition and dynamics and in blood–CSF barrier function [2–13]. This indicates that studying CSF profiles may be helpful in distinguishing clinically related but immunopathogenetically distinct diseases.

To address the question of whether MDS is just a very active and tumefactive variant of MS or an immunologically distinct disease entity, we set out to systematically compare the CSF features of MDS with those of MS.

## Methods

We performed a systematic review of all MDS cases published in English, German, French or Spanish in journals indexed in the PubMed database of the US Library of Science at the US National Institutes of Health between January 1960 and December 2018. Relevant publications were identified using the following search expression: (“Schilder” OR “Schilder’s” OR “myelinoclastic diffuse sclerosis” OR “diffuse myelinoclastic sclerosis” OR “periaxialis diffusa”) NOT Schilder[author]. Further papers were identified from the references lists of the papers retrieved. Exclusion criteria were a diagnosis of subacute sclerosing panencephalitis (SSPE), a diagnosis of adrenoleukodystrophy (ALD), abnormal very long-chain fatty acid (VLCFA) serum test results, abnormal adrenal function tests or familial CNS demyelination. Overall, we identified reports on 92 lumbar punctures in 66 individual patients.

The parameters assessed included, alongside epidemiological and clinical data, the presence or absence of CSF-restricted OCBs; OCB patterns; CSF IgG concentration and CSF/serum ratio (QIgG); Link’s IgG index ([IgG CSF/IgG serum]/[albumin CSF/albumin serum]); CSF white cell count; CSF cytology; CSF gamma-globulin fraction (γGF; as % of total CSF protein); measles/rubella/zoster (MRZ) reaction, as defined by a positive reaction to at least two of the three viral agents; CSF total protein (TP), glucose and lactate concentrations; CSF myelin basic protein (MBP) concentrations; CSF opening pressure; Epstein–Barr virus (EBV) and tuberculosis status; aquaporin-4 (AQP4) and myelin oligodendrocyte glycoprotein (MOG) antibody serostatus; VLCFA serum ratio; and adrenal function test results.

A CSF white cell count > 5/μl was classified as increased. An age-dependent upper reference range for CSF L-lactate was applied (0–15 years of age, 1.8 mmol/l; 16–50, 2.1 mmol/l; > 50, 2.6 mmol/l). The upper reference limit for CSF TP was set at 45 mg/dl, for CSF IgG at 6 mg/dL, for IgG index at 0.7, for γGF at 13% [14], for CSF glucose at 50% of serum glucose or 80 mg/dl and for CSF opening pressure at 250 mmH_2_O.

Subgroup analyses were performed for patients ≥ 18 years of age at onset (adult onset subgroup), patients < 18 years of age at onset (non-adult onset subgroup) and patients with available VLCFA results. Data from the MDS cohort were compared to publicly available data from the MSBase registry [15] and to data from reference studies in MS [16–21]. Differences in frequency distributions among groups were studied using Fisher’s exact test. Due to the exploratory nature of this study, no corrections for multiple testing were made. All analyses were performed retrospectively; accordingly, no CSF or serum samples were obtained for this study.

## Results

### Patient characteristics

The sex ratio (male:female) was 1:1.13, which is slightly lower than that in classic MS according to data from the MSBase registry [15] (1:2.39; *N* = 61,889). The median age at onset was 12 years (range 2–69) in the total cohort, 17 years (range 6–69) among females (*N* = 35) and 10 (range 2–45) among males (*N* = 31). This is in contrast to classic MS, in which the median age of onset is around 30 years (MSBase registry [15]; *N* = 61,889; *p* < 0.00001). The patient’s genetic background was not specified in most cases. Patients were reported by authors affiliated to centres in Europe (*N* = 34; Austria, France, Germany, Hungary, Italy, Poland, the Netherlands, Spain, UK), North America (*N* = 12; Canada, USA), the Middle East (*N* = 9; Iran, Israel, Turkey), the Far East (*N* = 3; China, Japan), South America (*N* = 1; Brazil) and Australia (*N* = 1). Further patients were of African (4 × South African, 1 × Moroccan) and Jewish (1 ×) descent. Exact data on the timing of lumbar puncture (LP) was insufficient or missing in most reports. However, LP was explicitly carried out after immunotherapy in only 1/82 (1.2%; no exact data in 10) and was performed during phases of active disease in at least 62/70 (88.6%; no exact data in 22). The disease course was described in most cases but not defined consistently across the various studies. At last follow-up, only a single (though often prolonged) attack had occurred in 26 patients; 24 had suffered two or more distinguishable episodes (median 3); and 11 had experienced progressive and continued deterioration. Median disease duration at last follow-up was 29 months (range 1–288).

### Oligoclonal IgG bands

OCBs were present in only 23% of all LPs (*N* = 39) tested for that marker and, at least once, in 26% of all patients tested (*N* = 35) (Table 1). The frequency of OCBs was slightly lower in the adult onset subgroup (18%; *N* = 11) than in the childhood onset subgroup (29%; *N* = 24), but the difference did not reach statistical significance (*p* = 0.65). This contrasts with a frequency of OCBs of 98% in adult MS and of 92% in childhood MS in two reference studies (*N* = 267 [17] and 136 [16], respectively) (*p* < 0.0001 and *p* < 0.0001; Fisher’s exact test, 2-tailed).

**Table 1:** Age and sex distribution, CSF OCB positivity rates, CSF WCC, CSF TP and CSF glucose and lactate in patients diagnosed with MDS. ^§^Assuming that the respective parameter was normal in samples classified as “normal” or “otherwise normal” (see the “Results” section for details). *Abbreviations*: *CSF* cerebrospinal fluid, *LP* lumbar puncture, *MDS* myelinoclastic diffuse sclerosis, *OCB* oligoclonal bands, *TP* total protein, *WCC* white cell count

	Total cohort			VLCFA-tested subgroup (VLCFA normal in all)		
	All	Adult-onset	Childhood-onset	All	Adult-onset	Childhood-onset
Sex ratio, male:female	1:1.13	1:2.67	1:0.76	1:1.17	1:2.33	1:1
Age at onset, median (range), years	12 (2–69)	29 (18–69)	9 (2–17)	10.5 (3–50)	32.5 (20–50)	8.5 (3–17)
OCB, positive, all LPs	23.1% (9/39)	15.4% (2/13)	26.9% (7/26)	14.3% (4/28)	0% (0/10)	22.2% (4/18)
OCB, positive, all LPs, including LPs classified as “normal” (1983–)	17.6% (9/51)	0% (2/18)	0% (7/33)	10.5% (4/38)	0% (0/14)	16.6% (4/24)
OCB, positive, first LP	21.9% (7/32)	10% (1/10)	27.3% (6/22)	13.6% (3/22)	0% (0/8)	21.4% (3/14)
OCB, positive, patients	25.7% (9/35)	18.2% (2/11)	29.2% (7/24)	16.7% (4/24)	0% (0/8)	25% (4/16)
WCC, elevated, all LPs	20.7% (12/58)	32.1% (9/28)	10% (3/30)	7.7% (2/26)	20% (2/10)	0% (0/16)
WCC, elevated, all LPs, including LPs classified as “normal”	15.2% (12/79)	26.5% (9/34)	6.7% (3/45)	4.9% (2/41)	14.3% (2/14)	0% (0/27)
WCC, elevated, first LP	25% (10/40)	43.8% (7/16)	12.5% (3/24)	10.5% (2/19)	28.6% (2/7)	0% (0/12)
WCC, elevated, patients	24.4% (10/41)	43.8% (7/16)	12% (3/25)	10.5% (2/19)	28.6% (2/7)	0% (0/12)
WCC, elevated, median (range), all LPs	25 (11–277; *N* = 11)	24 (12–277; *N* = 8)	32 (11–36; *N* = 3)	150 (23–277; *N* = 2)	150 (23–277; *N* = 2)	N.a. (N.a.; N = 0)
WCC, elevated, median (range), first LPs	25 (11–277; *N* = 9)	24 (12–277; *N* = 6)	32 (11–36; *N* = 3)	150 (23–277; *N* = 2)	150 (23–277; *N* = 2)	N.a. (N.a.; *N* = 0)
WCC, elevated, median (range), patients	25 (11–277; *N* = 9)	25 (15–277; *N* = 6)	32 (11–36; *N* = 3)	150 (23–277; *N* = 2)	150 (23–277; *N* = 2)	N.a. (N.a.; *N* = 0)
TP, elevated, all LPs	43.8% (28/64)	46.4% (13/28)	41.7% (15/36)	37.5% (12/32)	40% (4/10)	36.4% (8/22)
TP, elevated, all LPs, including LPs classified as “normal”	32.9% (28/85)	38.2% (13/34)	29.4% (15/51)	26.1% (12/46)	28.6% (4/14)	25% (8/32)
TP, elevated, first LP	43.8% (28/64)	46.4% (13/28)	41.7% (15/36)	37.5% (12/32)	40% (4/10)	36.4% (8/22)
TP, elevated, patients	41.3% (19/46)	43.8% (7/16)	40% (12/30)	37.5% (9/24)	42.9% (3/7)	35.3% (6/17)
TP, elevated, median (range), all LPs	114 (46–220; *N* = 22)	135 (46–182; *N* = 11)	112 (56–220; *N* = 11)	103 (46–212; *N* = 7)	105.5 (46–165; *N* = 2)	103 (56–212; *N* = 5)
TP, elevated, median (range), first LPs	116 (46–220; *N* = 15)	97.5 (46–180; *N* = 6)	116 (63–220; *N* = 9)	134 (46–212; *N* = 6)	105.5 (46–165; *N* = 2)	134 (63–212; *N* = 4)
TP, elevated, median (range), patients	107.5 (46–220; *N* = 18)	60 (46–182; *N* = 7)	112 (56–220; *N* = 11)	103 (46–212; *N* = 7)	105.5 (46–165; *N* = 2)	103 (56–212; *N* = 5)
Glucose, normal, all LPs	85% (17/20)	80% (4/5)	86.7% (13/15)	100% (14/14)	100% (4/4)	100% (10/10)
Glucose, normal, all LPs, including LPs classified as “normal”	93.9% (46/49)	92.9% (13/14)	94.3% (33/35)	100% (33/33)	100% (11/11)	100% (22/22)
Lactate, normal, all LPs	100% (7/7)	100% (1/1)	100% (6/6)	100% (7/7)	100% (1/1)	100% (6/6)
Lactate, normal, all LPs, including LPs classified as “normal”	100% (35/35)	100% (10/10)	100% (25/25)	100% (24/24)	100% (8/8)	100% (16/16)

If only patients in whom ADL was excluded by a negative VLCFA test result are considered ('high diagnostic certainty subgroup'), the frequency of CSF-restricted OCBs was as low as 14% (4/28). When applying even stricter criteria by taking into account only cases in which both VLCFA and adrenal function test results were available and normal, no patient had OCBs.

Of the few patients (*N* = 12) in whom an inflammatory disease was suspected but ALD not excluded (no VLCFA and no adrenal test results available) and in whom neither female gender nor favourable outcome argued against ALD ('ALD risk group'), none had been tested for OCBs. In consequence, these patients were not considered when calculating OCB rates. Therefore, accidental inclusion of patients with ALD is unlikely to have contributed greatly to the low rate of OCBs in MDS observed in this study.

In some case reports, CSF was classified as “normal” (or “otherwise normal” if reporting was limited to pathological items) but it was not specified whether OCBs were tested or not. Given that OCBs are included in both the Poser criteria for MS and the 1999, 2001 and 2017 McDonald criteria for MS and are therefore more or less routinely tested in patients presenting with CNS demyelination, it is likely that some of the CSF samples classified as “normal” or “otherwise normal” were tested also for OCBs. This would imply that the true frequency of OCBs was even lower than calculated from the available data. Assuming OCB negativity in the samples that were classified as  “normal” or “otherwise normal” and tested since inclusion of OCBs in the diagnostic criteria for MS in 1983, the OCB rate would be as low as 17.6% in the total cohort and 10.5% among VLCAF-tested patients.

Almost all positive OCB results were obtained between 1983 and 2003; only a single patient tested between 2004 and 2018 (*N* = 11) was positive for OCBs. This argues strongly against lower sensitivity of isoelectric focusing or OCB staining methods in the past accounting for the low rate of OCBs in MDS in the literature.

### CSF white cell counts

CSF white cell counts (WCC) were elevated in only 21% (12/58) of all LPs and in only 24% (10/41) of all patients (Table 1). They were particularly low in the pediatric subgroup, in which pleocytosis was found in only 10% (3/30) of all samples and, at least once, in 12% (3/25) of all patients. This contrasts with a pleocytosis rate of over 50% in patients with MS (*p* < 0.0002 vs. [18]). The median WCC in CSF samples with pleocytosis and available data was 25 cells/μl (range 11–277; *N* = 11; < 40 in 10/11 [90.9%]. Cytology results were reported for 9/12 patients with pleocytosis; only lymphocytes were found in five of those cases; lymphocytes and monocytes in one; lymphocytes and less than 20% granulocytes in one; and predominantly polymorphonuclear cells in the only patient with more than 40 cells/μl.

In the subgroup of patients who were tested for VLCFA ('high diagnostic accuracy subgroup'), pleocytosis was noted with only 8% (2/26) of the LPs. Of particular note, CSF pleocytosis was absent in all LPs from VLCFA-tested patients in the childhood onset subgroup (*N* = 16).

In some reports, LP findings were classified as “normal” or “otherwise normal” by the authors. Given that WCC assessment is usually done in patients undergoing LP, the true frequency of pleocytosis may thus be even lower. Assuming that WCC was normal in those instances, the rate of LPs with pleocytosis was just 5% in the VLCFA-tested subgroup.

### CSF total protein, lactate and glucose

Elevated CSF TP levels were present in at least 28/64 (44%) of all LPs with available data and at least once in 23/47 (49%) patients, with no marked difference between adults (13/28; 46%) and children (15/36; 42%) (Table 1). This contrasts with a TP elevation rate of 23.3% in 407 patients with MS as observed in a recent study [19] (*p* < 0.0001).

The median TP concentration in patients with pathological values was 114 mg/dL (range 46–220; *N* = 22; no exact data available in 6 cases), and TP values exceeded 100 mg/dL in 13/22 (59%). By contrast, TP levels over 100 mg/dL are rare and, according to a consensus paper, should prompt a search for alternative diagnoses in patients with suspected MS [20]. Similarly, the maximum TP concentration observed in a cohort of 111 paediatric MS patients was just 72 mg/dL.

CSF lactate levels were reported in only 7 patients and were normal in all. CSF glucose levels were reported for 20 LPs in 18 patients and were slightly elevated in 2; a decrease in CSF glucose was reported in none of the patients.

### MRZ reaction, EBV and tuberculosis

The MRZ reaction was tested in five LPs from four patients diagnosed with MDS and was negative in all. In one other case, CSF and serum antibodies to measles and rubella were “not elevated”; in two additional cases, antibodies to measles were absent or “present only at low titer”, respectively, in CSF and serum; and in two further patients, “serological titers to all common viruses” or “against neurotropic viruses”, respectively, were negative. Finally, one patient had CSF antibodies to measles and varicella zoster virus, but it is unclear from the report whether the antibody was produced intrathecally (as required for diagnosing a positive MRZ reaction) or had entered the CSF from the periphery (as is the case also in healthy individuals).

Serum antibodies to EBV, which are present in virtually all patients with MS, were tested in at least five patients and were negative in all.

Interestingly, MDS was associated with tuberculosis in three of seven children with available data (3 × exposure to adults with tuberculosis, including two cases with strongly positive Mantoux skin test): two of African descent and one described as “coloured”.

### Other parameters

Link’s IgG index was positive in two additional, OCB-negative patients. However, Link’s index has been shown to be prone to false-positive results in patients with blood–CSF barrier (BCB) dysfunction, due to the use of a linear instead of a hyperbolic reference curve, and is therefore no longer considered a reliable marker of intrathecal IgG synthesis [22]. Indeed, in both cases high TP CSF levels (165 and 103 mg/dL) were noted indicating possible BCB dysfunction (albumin ratios not reported). In consequence, the validity and relevance of IgG index elevation remains unknown in these cases.

A slightly elevated γGF was noted in 2 patients (13.8% and 14%, respectively; upper reference limit 13%), including one of the OCB-positive patients, while γGF was normal in 11 further LPs. In common with the IgG index, γGF is an unreliable and thus obsolete marker of intrathecal IgG synthesis. Data on IgG CSF/serum ratios and Reibergrams, two better established markers of intrathecal IgG synthesis, were reported in no patients and only one patient (demonstrating absence of IgG synthesis), respectively.

CSF MBP concentrations were measured in four patients and were elevated in three of them (207 ng/ml [normal < 4] in one, not specified in the remainder). This contrasts with mean CSF MBP levels of 8.2 ng/ml in monosymptomatic and 22.3 ng/ml (elevated in 95%) in polysymptomatic MS in a reference study [21].

Serum AQP4-IgG antibodies were reported in two cases and were negative in both [23]. MOG-IgG was reported in none. CSF opening pressure was measured during 12 LPs in 11 patients and was elevated in two; in these cases, the opening pressure was 280 and 510 mmH_2_O, respectively.

### CSF findings in Schilder’s original case

For the sake of completeness, it should not go unmentioned that Schilder’s index patient also underwent LP. Overall, four CSF samples were obtained within 3 months. The CSF WCC was normal in the first sample (although the sample was analysed only 24 h after LP) taken 1 day after admission and 8 weeks after symptoms started, was slightly elevated at day 15 (“5-8 cells per field of view”), not assessed in the third sample and normal at day 70 despite most severe neurological deterioration. TP was tested only in the second sample and was slightly elevated. The CSF opening pressure was assessed on the occasion of the second (200 mmH_2_O), third (350 mmH_2_O) and fourth puncture (“*Liquor unter starkem Druck*” [CSF under strong pressure]). While the Wassermann reaction (a test indicating a possible history of syphilis) was positive in the serum at admission, it was—after treatment with arsphenamine (salvarsan) for suspected neurosyphilis—negative twice in the CSF. However, when the CSF was tested using Stern’s modified Wassermann test, it was positive.

### Clinical and paraclinical findings in the OCB-positive subgroup

In seven of the nine OCB-positive patients, the disease had started during childhood or adolescence (at ages 5, 7.5, 9, 10, 12.5, 15 and 17 years) and in two during adulthood (at ages 18 and 24 years). The sex ratio (m:f) was 1:3.5 and thus slightly higher than that in the total cohort (1:1.13). At last follow-up, one patient had experienced only one attack (median follow-up, 4 years since onset) with almost complete recovery, while six patients had experienced at least two attacks (follow-up period 8 months, 19 months, > 19 months, 2 years, 4 years and 8 years), with complete recovery in one, almost complete recovery in three and fatal outcome in the remaining two, who died during a tonic-clonic seizure and of pneumonia following severe neurological deterioration, respectively. In two other patients, the disease took a progressive course resulting in severe aphasia, cognitive deficits and paresis after two years in one of them and leading to death after 3.25 years in the other. ALD was not formally excluded by VLCFA measurements in 5/9 cases (as stated above, after exclusion of patients not tested for VLCFA, only 17% [4/24] were OCB positive). However, all of those five patients were females, which renders ALD unlikely. Moreover, adrenal function tests were normal in two (and, in addition, adrenal autopsy in one of them). In one of the two patients with no VLCFA or adrenal test results available, the favourable outcome with complete recovery argues against ALD; in the other, at least basic plasma cortisol values and urinary 17-ketosteroids were tested and found to be normal. No information on OCB patterns [22, 24] was given. CSF WCC were reported in four and were elevated in two (1 × “slightly elevated”, 1 × 32 cells/μl), with cytology revealing lymphocytes (2 ×) and monocytes (1 ×); in two other patients, CSF was reported to be “(otherwise) normal”, suggesting that WCC were normal. CSF TP was elevated in three of six patients (no data in two). Six of the nine OCB-positive patients met the current diagnostic criteria for relapsing–remitting MS (RRMS), one for primary progressive MS [25]; a further patient presented with a focal seizure half a year after first presentation but showed no new lesion; and one patient had experienced visual loss 3 years before diagnosis, but the information about that episode was not sufficient to decide whether the criteria for RRMS were met. As a limitation, it should be stressed that clinical data were sparse in some reports, leaving some doubt regarding the exact clinical course. In one OCB-positive case, the spinal cord was involved as well. CSF MBP concentrations were measured in one patient and were elevated. In seven cases, histological data from biopsy (4 ×) or autopsy (3 ×) samples were available; the findings were generally compatible with a diagnosis of MDS. However, sparing of U-fibers as noted in Schilder’s index case (but rare in classic MS) was mentioned only in three of the OCB-positive cases and was incomplete in two of them (“rarely spared”; “U fibers involved in some areas”). No exact data on the patients’ genetic background were given: one was from Brazil (“dark-skinned”), one from Morocco, one from Turkey, one from the USA and five cases were reported by European-based centres (2 × Spain, 1 × Germany, 1 × Hungary, 1 × Italy). All LPs demonstrating positive OCBs were performed between 1983 and 2003.

## Discussion

Schilder’s myelinoclastic diffuse sclerosis (MDS), Devic’s neuromyelitis optica (NMO) and Balo’s concentric sclerosis (BCS) have all been described as “variants of MS” for many decades. However, research performed over the past two decades has cast serious doubt on the traditional idea of MS being a homogeneous disease with a number of clinicoradiological “variants”:

1. The demonstration of substantial histopathological as well as immunological differences among biopsied or autopsied patients with clinicoradiologically defined bona fide MS ('pattern 1 MS', characterized by T cell and macrophage infiltration, vs. 'pattern 2 MS', defined by additional antibody and complement deposition, suggesting a contribution of humoral mechanisms to disease pathology, vs. 'pattern 3 MS', characterized by distal oligodendrogliopathy with dysregulated myelin protein expression and oligodendrocyte apoptosis, still occuring on an inflammatory background) has reintroduced the idea of immunopathogenetic heterogeneity among patients with inflammatory CNS demyelination [8, 26, 27].
2. AQP4-IgG-positive NMO has been convincingly shown to be distinct from MS with regard to pathogenesis, prognosis and optimum treatment and—after a short period of disbelief and debates between “lumpers and splitters” [28]—is now considered a disease entity in its own right by virtually all experts in the field.
3. MOG-IgG-positive encephalomyelitis (EM), which shares substantial clinical overlap with both MS and NMOSD, has also recently been shown to be an immunologically distinct entity in its own right [29].
4. The recent identification of novel autoantibodies against, for example, glial fibrillary acidic protein (GFAP) [30] or the flotillin-1/2 heterocomplex [31] in patients with CNS demyelination and reports on demyelination in patients with anti-*N*-methyl-d-aspartate receptor (NMDAR) encephalitis [32] have the potential to further challenge the idea of MS as an immunologically homogeneous disease in the future.
5. Finally, recent studies [33, 34] suggesting substantial differences between MS and BCS in regard to both radiological and histopathological presentation and immunopathology have meanwhile also cast doubt on the concept of BCS being a “variant of MS”.

In the present study, we demonstrate significant differences in CSF pathology between MS and MDS. Our findings suggest that MS and MDS may be immunologically distinct entities in most cases. Most strikingly, only 26% of all patients diagnosed with MDS and only 17% of those with a normal VLCFA test had OCBs. The fact that no significant difference in the frequency of OCBs was noted between 'historic' and more recent MDS patients, with only a single case of OCB-positive MDS reported since 2003, widely rules out the possibility that lower assay sensitivity in the past played a major role. The low frequency of OCBs in MDS is in stark contrast to MS, in which OCBs are considered a diagnostic hallmark. In addition, we also found a significant difference between MS and MDS with regard to the frequency of CSF pleocytosis. Finally, in many cases, CSF TP concentrations in MDS were much higher than typically seen in MS.

**Fig. 1:**
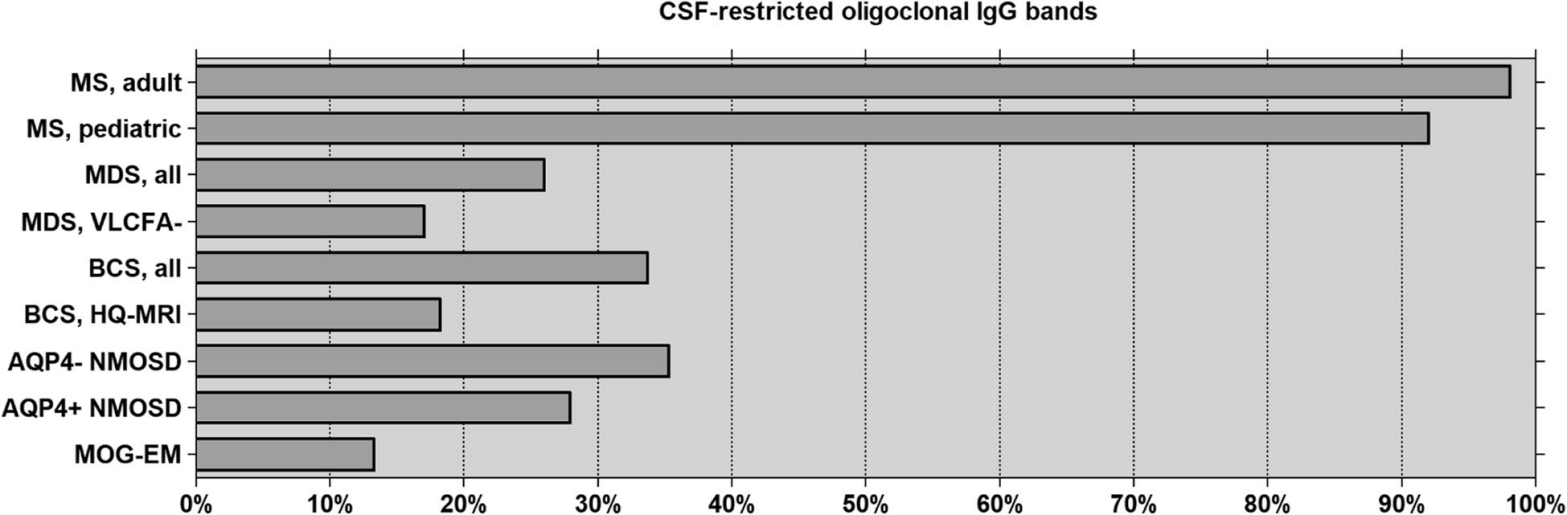
Frequency of CSF-restricted OCBs in patients with adult MS (*N* = 276) [18], pediatric MS (*N*=136) [16], MDS (*N* = 35; present study), VLCFA-negative MDS (*N* = 24; present study), BCS (*N* = 146) [32], NMOSD (*N* = 144) [3] and MOG-EM (*N* = 45) [10, 11, 34, 35]. AQP4+ aquaporin-4-IgG-positive, AQP4− aquaporin-4-IgG-negative, BCS Baló’s concentric sclerosis, HQ-MRI high-quality MRI subgroup (see the “Results” section for details), MDS myelinoclastic diffuse sclerosis, MOG-EM myelin oligodendrocyte glycoprotein antibody-positive encephalomyelitis, MS multiple sclerosis, NMOSD neuromyelitis optica spectrum disorders, VLCFA− subgroup tested for very long-chain fatty acids (normal in all)

We have already demonstrated that CSF profiles differ significantly between MS and NMOSD [2, 3], MS and BCS [33], MS and MOG-EM [10, 11, 35, 36] and between biopsied/autopsied patients with suspected MS and so-called pattern 1 brain lesions on the one hand and patients with so-called pattern 2 or 3 brain lesions on the other [8] (Fig. 1). Given these differences in CSF pathology, which include discrepancies in hallmark features of MS, it seems likely that NMOSD, BCS, MOG-EM and MDS are entities immunologically distinct from MS in the majority of cases. In the future, therefore, we recommend abstaining from generally referring to NMO, BCS, MOG-EM and MDS as “variants of MS”—a wording still found in many neurological textbooks and in many review articles on demyelinating diseases of the CNS. While those disorders seem all to be inflammatory in nature and to result in primary (as in MS and MOG-EM) or secondary (as in NMOSD and possibly BCS) demyelination, they may not share a common immunopathogenesis.

Correct classification and differentiation of diseases is of the utmost importance when it comes to clinical trials. Treatment recommendations in MS are based on large phase III studies that probably included, as suggested by prevalence, mainly patients with conventional MS. It is a priori unknown whether the treatments found to be effective in such studies are equally effective (or effective at all) in demyelinating disorders that share clinical features but are pathogenetically distinct. The recent reclassification of NMOSD as a discrete disease distinct from MS has prompted studies that have reviewed treatment outcome in NMOSD and in fact have revealed substantial differences in therapy response and optimum treatment between the two conditions: interferon beta and a number of other MS drugs have been found to be probably ineffective or even detrimental in NMOSD [37] (while others, such as B-cell depleting drugs, may be effective in both disorders), and preliminary studies indicate that the same may hold true for MOG-EM [10, 35].

Currently, there is no evidence-based therapy for MDS. However, several case reports suggest that (high-dose) steroid treatment may be effective. Other treatments such as IVIG or rituximab have been tried in single patients only, with no definite data on efficacy being available. The use of immunomodulatory drugs approved for the treatment of MS may be considered in selected patients, e.g. in patients with suspected MDS but later conversion to definite MS, especially if OCB-positive, and in borderline cases suggestive of either MDS or MS. That said, more data are certainly needed before any definite recommendations on the treatment of MDS can be made.

### Historical perspective

From a historical perspective, it is interesting to learn that Eugène Devic (1858–1930), the eponym of Devic’s disease [38], i.e. NMO, Joseph Baló (1895–1979), eponym of Balo’s disease, i.e. BCS, and Paul Schilder, eponym of Schilder’s disease, i.e. MDS, were all rather hesitant in classifying the conditions they encountered in their patients as MS. However, the opinion of later authors who had a broader audience finally prevailed. While Devic and his pupil Fernand Gault (1873–1936) had underlined in 1894 that lesion morphology in NMO differs substantially from that in MS [39], most textbooks of the 20th century would hold the view that NMO was a “variant of MS”, an opinion that can be traced back at least to the 1930s. Lord Brain (1895–1966), long-time editor of *Brain* and principal author of *Brain’s Diseases of the Nervous System*, the standard textbook of its time, stated in a widely cited 1930 review article on disseminated sclerosis that “The clinical and pathological differences between neuromyelitis optica and disseminated sclerosis appear to be differences of acuteness and intensity only (…) [currently] there seems no justification for separating them” [40]*.* Nowadays, NMO (or at least AQP4-IgG-positive NMOSD) is recognized as a disease entity in its own right by virtually all experts in the field [37, 41–43]. Similarly, Baló had believed his cases to represent a “disease [that] differs from multiple sclerosis” as well as from Marburg’s “acute MS” and Schilder’s “encephalitis periaxialis diffusa”, and which he proposed to term “leuko-encephalitis periaxialis concentrica” [44]. However, he could not prevent BCS later being defined as a “variant of MS” by some of the most authoritative textbooks right up to the modern day. Likewise, Schilder concluded in his 1912 article that the differences in clinical presentation were “*hinreichend ausgeprägt, um wenigstens vorläufig die Encephalitis* [*periaxialis*] *diffusa der multiplen Sklerose als selbständiges Krankheitsbild gegenüber zu stellen* [pronounced enough to allow, for the time being, contrasting encephalitis diffusa with multiple sclerosis as a self-contained disease entity]” [1], only to be contradicted later on by a figure as eminent as Charles M. Poser (1923–2010), lead author of the 1983 diagnostic criteria for MS, who in 2004 [45] would still maintain the view that there was “little doubt” that MDS was “simply another form of MS” (see also Poser's "revised classification of the inflammatory demyelinating diseases" in [45]). Based on OCB frequency, we believe that this may not be correct, at least in the majority of cases.

Readers interested in the history of neurology may appreciate to learn that Poser and Bogaert, in their seminal 1956 article on MDS (in which they pointed out that the cases described by Schilder in 1913 [46] and 1924 [47] rather represented instances of leukodystrophy and subacute sclerosing encephalitis) classified Devic’s NMO as a variant of Schilder’s MDS [48], thereby further contributing to nosological confusion. The only patient with MDS so far tested for serum AQP4-IgG, a highly specific marker of NMO, was negative for that marker [23] (a second patient was negative for CSF AQP4-IgG [49]; however, CSF is not the specimen of choice when it comes to testing for AQP4-IgG [50]).

### Further differences between MDS and MS

In addition to the significant differences in terms of oligoclonal IgG synthesis and cellular CSF immune response described above, we would like to draw attention to several further lines of evidence suggesting that MDS may not be the same disease as MS, with special reference to Schilder’s index case (a previously healthy 14-year-old prepubescent girl with eight healthy siblings).

#### 1. Differences in lesion size and distribution:

(a) While MS (“encephalomyelitis disseminata”) is typically characterized by a multitude of disseminated, more or less circumscribed lesions, Schilder’s patient showed a large contiguous demyelinating lesion affecting most of the white matter of the right hemisphere and the entire corpus callosum and even extending through the corpus callosum to the white matter of the other hemisphere. Schilder considered this difference highly significant (“*ein sehr wesentliche[r Unterschied] […] [zur] gewöhnlichen Multiplen Sklerose*”).

(b) MS frequently affects the spinal cord from earliest stages on [51]. By contrast, spinal cord inflammation was described in only a few of the patients diagnosed with MDS in the present study (one of whom was positive for OCBs, had a second attack and met the clinicoradiological criteria for RRMS, which casts some doubt on the diagnosis). Spinal cord lesions were also absent in Schilder’s index case, as were lesions in the brainstem, cerebellum and medulla oblongata, areas often affected in MS (“*Am Kleinhirn, am Hirnstamm und der Medulla oblongata bestehen pathologische Veränderungen nicht. Auch Durchschnitte durch das Rückenmark sind in keiner Weise auffällig*”).

(c) Neuropathologic and neuroimaging studies have demonstrated involvement of the basal ganglia and thalamus in patients with MS [52–55]. By contrast, the basal ganglia were spared in Schilder’s case, as mentioned thrice in his article: “*[Der Prozess] greift nicht auf die basalen Ganglien über […] macht an […] den großen Ganglien halt […] die basalen Ganglien zeigten keine Veränderungen*”. As a limitation, the patient’s young age could have played a role [56].

(d) While the cortex was almost completely spared in Schilder’s patient despite the extensive damage in the white matter, it is frequently affected in MS [57, 58]. The latter fact was already known to Schilder: *“Es verschonen also die Herde der multiplen Sklerose die Rinde nicht* [lesions in MS do not spare the cortex]”, as he stated with reference to the findings of Otto Marburg (1874–1948) and Herrmann Oppenheim (1857–1919). For the sake of completeness, it should not go unmentioned that Schilder observed a single, very small area of cortical damage. However, according to Schilder, the lesion was placed at a *locus typicus* for cortical insult after Bramann–Anton’s “*Balkenstich*”, an operation performed in his patient exactly 3 months prior to autopsy. The operation was personally carried out by Friedrich Gustav von Bramann (1854–1913), and Gabriel Anton (1858–1933), in collaboration with whom the method had been developed, proofread Schilder’s article before publication (as can be seen from the acknowledgement), suggesting that Schilder’s hypothesis in this regard was somewhat 'evidence-based'. Regarding that small cortical lesion, Schilder noted several differences from MS lesions as well as from his patient’s large white matter lesion: the process was markedly destructive (“*auffallend destruktiv*”, “*beträchtlich schwerer als die des Marks*”), also in the non-cystic parts, with non-preservation of the axons, destruction of the neuronal bodies, strong proliferation of small vessels and severe pial damage.

(e) Schilder stressed that the U-fibers were spared in his patient (“*macht an den Fibrae arcuatae halt*”). This is of interest in the light of recent studies demonstrating absence of U-fiber lesions in 20/21 (95.2%) patients with MOG-EM in a mixed adult (*n* = 15) and paediatric (*n* = 6) cohort [59, 60] as detected by MRI. Similarly, U-fiber lesions were absent in 65/69 (94.2%) MOG-IgG-positive patients in a paediatric cohort [61]. By contrast, U-fiber lesions are frequently seen in MS, and juxtacortical lesions were included in both the 1997 Barkhof criteria for MS and the McDonald criteria for MS. Of note, preservation of the U-fibers has also been observed in patients with hereditary leukodystrophies, including X-linked ALD (at least until a far-advanced stage) and small-vessel diseases [62, 63]. U-fibers differ from other myelinated fibers with regard to age of myelination, myelin metabolism and turnover, and blood supply.

(f) The sparing of cortex, basal ganglia and U-fibers was explicitly considered by Schilder as a feature distinguishing his case from MS: “*Eine derartige Elektivität kommt bei der multiplen Sklerose nicht vor* [such electivity does not occur in MS]”.

(g) Schilder noted demyelination also in the optic chiasm, which is affected only rarely in MS, but commonly in AQP4-IgG-positive NMOSD [64]. However, this finding may be of limited relevance, since Schilder could not come to a definite conclusion as to whether the relatively mild demyelination observed was primary or rather secondary due to compression caused by increased brain pressure.

#### 2. Differences in histopathology:

(a) While small cysts may occur also in MS, the strongly cystic formation of parts of the white matter lesion in Schilder’s case, to which he referred as “*Mikrozysten*”, “*Zysten*”, “*Hohlräume*”, “*Höhlenbildung*”, “*Zerfallshöhlen*” [i.e. microcysts, cysts, cavitations, necrotic cavitations] and the structure of which he compared to honeycombs (“*eigenartig feinwabig gebautes Gewebe*”), would be unusual. Schilder considered this difference most important: “*Auf die Differenzen [zur MS] im histologischen Bild habe ich ja bereits verwiesen. Es sind vorwiegend die schweren degenerativen Erscheinungen am Gliagewebe. Der makroskopische Ausdruck dieser Differenz ist die Höhlenbildung*” [I have already pointed out the differences [from MS] in histological presentation. These are mainly the degenerative changes of the glial tissue. The macroscopic equivalent of that difference is the cavitation]. Interestingly, Poser gave a very similar description of lesion formation in a patient with MDS he had personally seen. In that patient, surgical exploration demonstrated “a firm mass made up of many microcysts with the general appearance of a beehive”. Histologically, the parenchyma around blood vessels “looked spongy and showed multiple, much interstitial edema-forming microcysts” [65]. Cystic structures were also described in other patients diagnosed with MDS in whom biopsy or autopsy were performed (e.g. [66–70]).

(b) In contrast to typical MS lesions, lymphocytes were almost completely missing and plasma cells completely missing in Schilder’s case, despite highly active disease leading to death after 5 months. This applies to all areas of the white matter lesion, including older and newer parts, the surrounding tissue, the (possibly unrelated) small cortical lesion and the pia mater. Even in the perivascular cuffs of most vessels only very rarely (“*nur ganz selten*”) were cells seen that could have been lymphocytes (although, alternatively, these few cells could have been reduced körnchenzellen according to Schilder). It should not remain unmentioned that Schilder also observed occasional small veins with somewhat more pronounced lymphocytic infiltration (“*etwas stärker ausgeprägter Lymphocyteninfiltration*”), though again without any evidence of plasma cells. However, he relativized that statement later in a summary of his findings by describing lymphocytes as being “*außerordentlich selten* [extraordinarily rare]”. Overall, he attached special importance to the contrast between the drastic tissue damage and the minor (“*geringfügig*”) degree of vascular inflammation as being one of the main differences of his case from typical MS. Inflammatory infiltrates were also absent in the demyelinated parts of the chiasm and optic nerve, including the vessel sheaths. As a limitation, plasma cells can be sparse in early-active MS lesions and their numbers are believed to vary among patients [71]. Of note, complete absence of plasma cells, which are the source of CSF-restricted OCBs, as well as sparing of the cortex and basal ganglia, have also been reported in Marburg’s acute 'variant of MS' [72]; in line with that finding, the patient had neither OCBs nor quantitative evidence of intrathecal IgG, immunoglobulin M (IgM) or immunoglobulin A (IgA) synthesis. However, in contrast to Schilder’s patient, patients with Marburg’s disease mostly have multiple lesions and—often fatal—brainstem lesions [72].

(c) Schilder mentioned a number of further circumstances he believed would argue in favour of his case being distinct from MS: markedly better preservation of axons than in MS; more pronounced nuclear alterations in glial cells; and more pronounced degradation of the large glial cells by microglial cells than seen in MS.

#### 3. Differences in associated infections:

(a) EBV has been involved in the pathogenesis of MS, with virtually all patients with MS being positive for serum antibodies against EBV [73, 74]. However, antibodies to EBV were absent in all five published patients diagnosed with MDS that were tested for that marker.

(b) Schilder’s patient as well as her mother showed a positive Wassermann reaction, which occurs in (congenital) syphilis, neurotuberculosis and systemic lupus erythematosus. Unfortunately, most later cases of MDS were not examined for syphilis (or results were not reported). Exposure to tuberculosis and/or a positive Mantoux test has indeed been described in 3/7 MDS patients with available data. Both neurosyphilis and neurotuberculosis are not normally associated with MS but are differential diagnoses of MS.

(c) Intrathecally produced polyclonal antiviral antibodies, which are probably part of non-specific bystander activation of intrathecal B cells, distinguish MS from other autoimmune disorders of the CNS: A positive intrathecal IgG immune response against measles, rubella and zoster virus (the so-called MRZ reaction, defined by positive antibody indices against at least two of those three viral agents) is the marker with the currently highest positive likelihood ratio for MS and has been shown to be detectable in around 70% of patients with MS [4, 18]. Intrathecal production of antibodies to measles (with or without concomitant antibodies against rubella and zoster virus) has been demonstrated in up to 86% of patients with MS and is the most common intrathecal antiviral immune response in MS, both in adults and in children [18]. However, all MDS patients tested had a negative MRZ reaction, and no indication for intrathecal IgG synthesis of antibodies against measles could be found in the present cohort.

#### 4. Differences in clinical presentation and outcome:

(a) Schilder’s patient died after 5 months of continuous deterioration. Her symptoms included, among others, severe psychiatric and neuropsychological symptoms and deficits (disorientation, dementia, apathy, somnolence, arrest of thought, selective dyslexia, acoustic neglect, pseudo-ophthalmoplegia), complete blindness, spastic hemiplegia with facial paresis, paresis of the other leg, neck stiffness and severe hyperpathia. In addition, signs of increased intracranial pressure (increased CSF opening pressure [up to 350 mmH_2_O], headache, nausea, repeat vomiting and, possibly, bilateral *stauungspapille*) were noted. Such a fulminant course is atypical for classic MS. Increased CSF opening pressure, which does not typically occur in MS, was also reported in some of the MDS cases evaluated for the present study (see the “Results” section for details).

(b) Definitive classification of the visual disturbances in Schilder’s case is difficult. Initially, temporal pallor indicated bilateral optic neuritis. In addition, prominent papilledema (up to + 5 dpt) was apparent in both eyes, indicating either papillitis or *stauungspapille* (i.e. a choked optic disk due to increased intracranial pressure [ICP]). Histologically, some degree of demyelination was noted in the chiasm and intracranial optic nerve. However, the patient also exhibited signs and symptoms of cortical blindness (visual anosognosia, persisting light reaction), in line with demyelination affecting virtually the complete occipital lobe. Bilateral optic neuritis leading to complete blindness within a few days is very uncommon in MS. However, it is a common feature of both MOG-EM and AQP4-IgG-positive NMOSD [10, 36]. Schilder also considered remarkable the fact that signs of optic nerve atrophy were seen just 11 days after onset, which is not typical for MS. Irrespective of whether the papilledema indicated increased ICP (*stauungspapille*) or inflammatory papillitis—Schilder himself remained equivocal in that regard, since the extracranial portion of the optic nerve was not examined histologically—or both, papilledema would be atypical in MS, which is not usually associated with increased intracranial pressure and mostly affects the retrobulbar parts of the optic nerves. Interestingly, papillitis was recently shown to be common in patients positive for MOG-IgG [10], a marker not yet tested in patients diagnosed with MDS.

### Limitations and strengths

We count the high number of LPs analysed (given the low prevalence of the disease) among the strengths of this study, together with the fact that our results were both highly significant and robust when retested in a subgroup with particularly high diagnostic certainty, i.e. after exclusion of patients not tested for VLCFA (see the “Results” section for details). With no differences between historic and recent patients in terms of OCB frequency and only very few patients having been treated at the time of LP, we can also broadly rule out an effect of assay sensitivity or treatment. Moreover, the very high rate of OCBs in classic MS is not a new finding linked to modern techniques but was reported in studies performed as long ago as some of the earliest MDS studies with available OCB data analysed here. Finally, so far an effect of immunotherapies on OCB positivity has been shown only for natalizumab [75, 76], which was not used in any of the patients analysed for this study.

On the other hand, the retrospective nature of our study and the fact that the patients included in the analysis were seen at many different centres are potential limitations. Given the condition’s very low prevalence, however, it is impossible to perform a large prospective single-centre study on MDS. Moreover, the study’s retrospective 'multicenter' setting reduced potential risks resulting from centre-specific selection bias, a problem inherent to single-centre studies. Second, no CSF results were given in several of the reports analysed for this study. This may have introduced a bias towards cases with pathological CSF findings. However, that would imply that our results actually underestimate the lack of OCBs and pleocytosis in MDS and would thus make our findings even more relevant.

The lack of generally accepted criteria for MDS or a gold standard laboratory marker is another potential limitation, which, however, cannot be avoided. By relying on the diagnoses made by the reporting physicians and providing a subgroup analysis of more recent cases, our findings relate to MDS as it is currently understood and diagnosed in clinical practice. In addition, we provide subgroup analyses for those patients in whom ALD was properly excluded in order to avoid any bias caused by unwittingly including patients with the metabolic form of MDS: OCBs were missing both in most patients with MDS in which ALD was properly excluded and in most patients with MDS in which ALD was not adequately ruled out. Patients with a diagnosis of ALD were not included at all.

The present study considered exclusively patients with a final diagnosis of MDS as made by the reporting authors. This could be considered a strength but also a potential limitation, since we may have overlooked further published cases of true MDS in which the authors did not arrive at the correct diagnosis. In 2009, Bacigaluppi and colleagues retrospectively identified 10 additional patients with possible MDS, based on clinical and paraclinical presentation, in whom the diagnosis of MDS was not made by the reporting authors (and who were thus not included in the present study) [77]. LP was performed in 9 of those 10 patients. It is reassuring that OCBs were absent in all of those cases too, confirming our findings. OCB negativity in MDS was also suggested by Tselis and Lisak in 2006, though based on retrospective analysis of only 6 cases [78].

While our data suggest that MDS and MS are different diseases in most instances, it is still possible that MS presents as MDS in some cases. Indeed, a number of similarities between MDS and MS exist: Both diseases result in demyelinating lesions of the CNS (though the spinal cord and the cerebral cortex—sites commonly affected in MS—seem to be mostly spared in MDS); both diseases may take a relapsing (e.g. [79]) or chronic progressive course (though monophasic disease seems to be common in MDS, but not in MS); perivascular lymphocyte infiltration (as seen in MS) has been reported in single cases (e.g. [80]); and large lesions (though not typically extending through the corpus callosum as reported in MDS) have been described to occur also in a small subset of patients with otherwise typical MS. It is thus theoretically possible that some patients classified as MDS had in fact MS, especially in the OCB-positive subgroup: At least six of the nine OCB-positive patients had experienced a second relapse, and seven met the current diagnostic criteria for MS (6 × RRMS, 1 × PPMS). Moreover, one of the OCB-positive patients additionally had spinal cord lesions, which are usually considered atypical for MDS, and at least one had optic neuritis, a common manifestation of MS. Finally, in 2003, Sastre-Garriga and colleagues indeed published an OCB-positive case of “Schilder-like onset” in otherwise typical MS [81]. That patient developed a new contrast-enhancing lesion after 6 months and left optic neuritis after 7 months. The final diagnosis was “clinically definite MS”. On the other hand, the observation time was quite long in most of the OCB-positive cases (up to 8 years; ≥ 2 years in 5/7; no data in 1), so that occurrence of new lesions, as usually seen in MS, should have been noted. In fact, a new lesion was observed in only two out of nine OCB-positive “MDS” patients. Moreover, the description of beehive-like lesion formation in Poser’s OCB-positive case matches Schilder’s original description better than that of typical MS lesions. It is therefore conceivable that OCB may indeed occur in a small subset of patient with true MDS. That would be similar to AQP4-IgG-positive NMOSD, BCS and MOG-EM, conditions in which OCBs are detectable in only 20–30% [2], 18–34% [33] and 12–13% [10] of cases, respectively.

Testing of patients with MDS for CSF-restricted OCBs, MRZ reaction and serum EBV-IgG might be helpful in differentiating MS and MDS. Given that Schilder’s original patient as well as the patient’s mother exhibited a positive Wassermann reaction and that MDS was associated with tuberculosis in some cases, those planning future studies in MDS should consider inclusion of testing for syphilis and tuberculosis in the diagnostic panel. Retrospective serological studies on MOG-IgG and AQP4-IgG in patients with MDS are currently being performed; however, testing for these markers in future patients with MDS is highly recommended.

The present study focused on immunological parameters routinely tested in patients with MS and MDS. Further immunological studies are now required to investigate the differences between MDS and MS in greater detail in terms of immunopathogenesis. Similarly, more endeavours are needed to better understand the relationship of MDS to other acquired demyelinating syndromes such as ADEM, MOG-EM and Marburg’s disease.

## Conclusion

This retrospective study strongly suggests that MS and MDS are immunologically distinct conditions by demonstrating highly significant differences regarding both the humoral and the cellular arm of the intrathecal immune response. We recommend hesitation in classifying MDS as a “variant of MS” in the future; rather MDS should be considered an entity immunologically distinct from classic MS in most cases. Our findings are not only of nosological relevance but also harbour potential therapeutic implications. Further studies are now needed to improve understanding of the differences in pathogenesis between MDS and MS suggested by our findings, as well as of potential differences in optimum treatment.

## Abbreviations

AQP4: Aquaporin-4
BCS: Baló’s concentric sclerosis
CNS: Central nervous system
CSF: Cerebrospinal fluid;
EM: Encephalomyelitis
IgA: Immunoglobulin A
IgG: Immunoglobulin G
IgM: Immunoglobulin M
LP: Lumbar puncture
MDS: Myelinoclastic diffuse sclerosis
MOG: Myelin oligodendrocyte glycoprotein
MRI: Magnetic resonance imaging
MRZ: Measles/rubella/zoster
MS: Multiple sclerosis
NMO: Neuromyelitis optica
NMOSD: NMO spectrum disorders
OCBs: Oligoclonal bands
TP: Total protein
WCC: White cell count
γGF: Gamma-globulin fraction (of total protein)
